# Weaning Outcome is Associated with ELWI and Impaired Diastolic Function

**DOI:** 10.2478/jccm-2024-0008

**Published:** 2024-01-30

**Authors:** Dimitra Bagka, George Zakynthynos, Vasiliki Tsolaki, Jonh Papanikolaou, Vasilis Vazgiourakis, Maria Baka, Konstantinos Pratsas, Demosthenes Makris

**Affiliations:** General University Hospital of Larissa, Larisa, Greece; University of Athens, Athens, Greece; General Hospital of Trikala, Greece

**Keywords:** weaning, EVLWI, diastolic dysfuction, critical care, mechanical ventilation

## Abstract

**Objectives:**

To evaluate hemodynamic profiles of critical care patients undergoing spontaneous t-piece trial (SBT) and present weaning failure.

**Methods:**

Prospective observational study conducted in ready-to-wean non-cardiac ICU patients. Clinical, echocardiographic and thermodilution-derived variables were recorded before and after a 2-hour SBT. Weaning from mechanical ventilation was defined as preservation of spontaneous breathing for 48 hours following successful SBT.

**Results:**

Fourteen patients succeeded weaning, five manifested T-trial-failure and six late-failure. Weaning outcome was significantly associated with ELWI(Extravascular lung-water index), global-end-diastolic index and impaired diastolic function, as indicated by pre-T Doppler early wave velocities (E/Em); Fifty-six percent of participants presented ELWI≥7mL/kg when fulfilling predetermined criteria for weaning. ELWI, impaired pulmonary permeability and left ventricular diastolic dysfunction were independent determinants of ELWI.

**Conclusions:**

ELWI before SBT and impaired diastolic function (as indicated by pre-T E/Em) might be weaning outcome determinants and their assessment may allow better risk stratification in weaning decision making.

## Introduction

Weaning from mechanical ventilation of critically ill patients is a challenging issue for critical care providers [1]. Even after meticulous initial screening for readiness-to-wean and successful T-piece weaning trials, failure rates may be considerably high [1]. This could be attributed to the complexity of the underlying pathogenic mechanisms involved in unsuccessful weaning.

Pulmonary edema complicating weaning has been increasingly emphasized as a mechanism that can lead to weaning difficulties and failure [1,2]. Patients with cardiac disease are particularly in risk for this complication [3,4]. However, extreme hemodynamic alterations in weaning may theoretically impair cardiac performance even in patients without underlying cardiac pathology [3].

We have previously shown that left ventricular (LV) diastolic dysfunction is associated with increased weaning failure rates in patients without pre-existing cardiac disease [5]. In the present study, we aimed to assess potential mechanisms related to pulmonary oedema during weaning trials in non-cardiac patients.

## Methods

This was a single-center prospective observational study. All mechanically ventilated patients who were admitted in the intensive care unit of a tertiary hospital between 2013–2015 were screened for eligibility and entered the study if they fulfilled the following inclusion criteria: clear improvement or resolution of the underlying condition for which the patient had been intubated based on treating physicians decision, hemodynamic stability (heart rate ≤110 beats·min^−1^, systolic blood pressure 90–160 mmHg, no vasopressors), minimal ventilator dependency (e.g. inspiratory oxygen fraction≤0.45, arterial oxygen tension ≥60mmHg, positive end-expiratory pressure≤8cmH_2_O), core temperature≤38.5°C, haemoglobin>7g/dL, alertness, ability to communicate, no need for sedation, placement of a thermodilution system (PiCCO) as part of their medical management based on treating physicians decision [1,6,7]. Exclusion criteria were: age <18years old, pregnancy, pre-existing or newly diagnosed heart disease (including LV ejection fraction<50% and/or LV wall abnormalities on echocardiography, non sinus heart rhythm, acute or chronic cor pulmonale of any etiology, preexisting cause of neuromuscular diaphragmatic weakness (i.e. myasthenia, muscular dystrophies, spinal cord injury, multiple sclerosis, amyotrophic lateral sclerosis, poliomyelitis, Guillain-Barre syndrome, diaphragmatic of phrenic nerve injury from tumor, trauma, following viral infection or radiation therapy), serum creatinine> 2mg/dL or need for renal replacement therapy or chronic renal failure. The protocol was approved by the institutional review board (No protocol 44363).

### Outcomes

The relationship between echocardiographic variables and hemodynamic indices obtained by thermodilution and weaning outcome was the primary objective of the study. Weaning from mechanical ventilation was defined as preservation of spontaneous breathing for 48 hours following a successful spontaneous breathing trial (SBT) was the primary outcome in this investigation. Secondarily, we sought to assess the relationship between ELWI and hemodynamic, echocardiographic indices.

### Study protocol

Patients underwent a 120-minute SBT and diagnostic tests were performed at three different time phases: i) while on assisted mechanical ventilation-pressure support (Pre-T measurements), ii) at 120 minutes of T-piece trial (End-T measurements) and iii) when signs of cardio-respiratory distress were present.

Clinical assessment, venous-arterial blood gas analysis using samples from a catheter placed in the superior vena cava, PiCCO hemodynamic measurements (Pulsiocath 5F, 20 cm, PV2015L20; Pulsion Medical Systems AG, Munich, Germany), echocardiographic examination (Phillips iE33, Andover, MA, U.S.A) and plasma BNP concentration (Biosite Triage® immunoassay, San Diego, CA, USA) were performed at the pre-specified time intervals. Clinical assessment included demographics, cause of admission, Acute Physiology and Chronic Health Evaluation II (APACHE II) score, Sequential Organ Failure Assessment (SOFA) score, daily fluid balance, creatinine level ≥2mg/dL, requirement for continuous renal substitution therapy, positive end-expiratory pressure (PEEP), rapid shallow breathing index (RSBI) [8], pressure frequency product (respiratory frequency x level of pressure support) (PFP) [9], maximal inspiratory pressure (MIP)[8], B-type natriuretic peptide (BNP) [10]. Pressure and flow transducers for hemodynamic assessment were carefully calibrated before starting each measurement which was taken at end-expiration. Echocardiographic studies were performed (by JP and VT) and analysed offline by investigators (VV and GZ) blinded to patients identities and hemodynamic measurements. Left ventricular ejection fraction (LVEF) was calculated from the apical 4-chamber view by using the Simpson’s method of disks, according to recommendations of American Society of Echocardiography and was classified as described [11].

### Definitions

A SBT was considered successful if no signs of cardio-respiratory distress [1] were observed within 2-hours following discontinuation from mechanical ventilation. Patients with orotracheal tubes with successful SBT were extubated. Weaning success was defined as the ability to pass the trial and remain on spontaneous breathing for more than 48 hours. Weaning failure was classified either as early-failure (unsuccessful SBT) or late-failure (within 48hrs following successful SBT). Patients remained in semi-recumbent position throughout the SBT. EVLW index (ELWI) ≥7mL/kg was used as a surrogate for pulmonary oedema [12].

### Statistical analysis

Results were expressed as means ± standard error (SE), unless otherwise stated. Chi-square or Fisher’s exact test were used to compare categorical variables and Student t-test or Man-Whitney U test to compare continuous variables, as appropriate. Linear regression analysis was used to determine associations among continuous variables and multivariate linear regression analysis was used to examine the effect of several univariate predictors in determining EVLW measurements independently. One-way analysis of variance was used for multiple comparisons. SBT induced hemodynamic and echocardiographic alterations between the success-group and failure-groups were compared over the time-course using repeated measures analysis of variance. Receiver operating characteristic curve analysis was performed to evaluate the diagnostic performance of echocardiographic variables in detecting pathologically elevated levels of ELWI. We considered P values of <0.05 (two-tailed) to be statistically significant. The statistical package SPSS 17.0 (SPSS Inc., Chicago, IL, USA) was used.

## Results

**Fig. 1.**
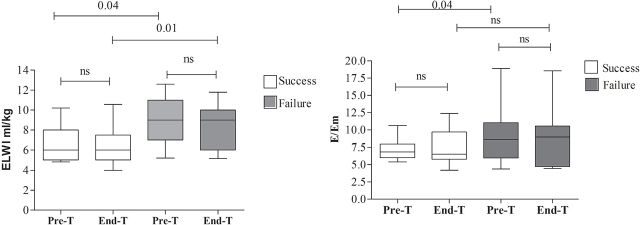
Extravascular lung water index (EVLWI) and E/Tissue Doppler Imaging early diastolic velocity (E/Em) in patients undergoing a spontaneous breathing trial before (Pre SBT) and following (End SBT) the trial, according to trial outcome (Median values, inter-quartile range and 10–90th percentiles are presented).

Twenty-five patients participated in the study. Patients’ clinical characteristics are presented in Table 1. Overall, fourteen out of 25 (56%) had successful weaning. PiCCO hemodynamics according to spontaneous breathing trial outcome before (Pre-T) and following (End-T) the trial are presented in Table 2. Weaning outcome was associated with ELVW (Figure 1); fourteen out of 25 (56%) patients presented baseline values of ELWI ≥7mL/kg. Weaning outcome was also associated with cardiac preload [global-end-diastolic index (GEDI)] (both Pre-T and End-T values) and the mean arterial pressure (MAP) (Pre-T values) (Table 2). Echocardiographic variables are presented in more detail in Table 3. ELWI values significantly associated echocardiographic variables are shown in Table 4. MIP End-T values were significantly higher in patients with successful weaning [26.1 (2.1) vs 20.4 (1.5), P=0.04].

**Table 1.** Baseline characteristics of participants according to spontaneous breathing trial outcome.

	**Weaning success (N=14)**	**Weaning failure**		**P-value**

		**EWF (<2h) (N=5)**	**LWF (2-48h) (N=6)**	
Age, years	58.31 (4.47)	63.25 (2.95)	67.83 (6.84)	0.45

Gender (male)	11 (78.5)	3 (60)	4 (66.7)	0.69

Medical /Surgical critical state	3 (21.5) / 11 (78.5)	2 (40) / 3 (60)	4 (66.7) /2 (33.3)	0.08
Sepsis	3	2	1	
Intoxication	0	0	1	
Pyelonephritis	0	0	1	
Status epilepticus	0	0	1	
Peritonitis	0	0	1	
Pancreatitis	1	0	0	
Neurosurgical	5	2	1	
Multiple trauma	2	0	0	
Cancer	1	1	0	
Hemorrhage	2	0	0	

Arterial Hypertension	9 (64.3)	5 (100)	5 (83.3)	0.24
COPD	1 (7)	1 (20)	0	0.46
Diabetes mellitus	3 (21)	1 (20)	0	0.47
APACHE II (on admission)	14.9 (2.66)	18 (4.37)	8.33 (5.84)	0.36
SOFA (on admission)	8.54 (1.64)	7.25 (1.65)	9 (1.46)	0.86
Fluid balance, mL/day	2676 (630)	472 (1667)	2513 (1564)	0.35
Renal dysfunction	3 (21)	2 (40)	2 (33.3)	0.69

**T-piece trial**				
ICU day of SBT	22.79 (6.06)	16 (6)	17.33 (2.65)	0.72
Orotracheal/Tracheostomy tube	11(78) / 3(21)	3(60) / 2(40)	4 (67) / 2(33)	0.69
SOFA	5.3 (0.9)	3.2 (1.1)	3.8 (0.7)	0.38
PaO_2_/FIO_2_ ratio	370 (31)	281 (78)	392 (36)	0.16
PEEP, cmH_2_O	6.3 (0.3)	7 (0.4)	6.7 (0.4)	0.52
*f*, breaths/min	19.7 (1.8)	24.2 (1.4)	21.1 (1.3)	0.24
V_T_, L	0.47 (0.014)	0.46 (0.017)	0.51 (0.026)	0.29
*f* /V_T_ (RSBI)	41.7 (3.9)	52.9 (4.7)	42.3 (3.7)	0.20
PFP, cmH_2_O_*_breaths/min	264 (201)	316 (27)	280 (27)	0.52
MIP, cmH_2_O	23.6 (1.57)	23.2 (1.83)	25.71 (1.83)	0.63
BNP, pg/mL	412 (157)	761 (728)	455 (125)	0.70
Albumin, mg/dL	2.7 (0.1)	3 (0.2)	2.8 (0.1)	0.32

Continuous data are presented as means±SE, categorical data as n (%).

* Spontaneous Breathing Trial-induced significant changes within subgroups (pre-trial vs, end-trial measurements), examined by paired t-test analysis

EWF= early weaning (T-trial) failure; LWF= late weaning failure; ANOVA=analysis of variance; COPD= chronic obstructive pulmonary disease; APACHE II=Acute Physiology and Chronic Health Evaluation Score II; SOFA score=Sequential Organ Failure Assessment score; PEEP= positive end-expiratory pressure; f= respiratory frequency; VT= tidal volume; RSBI= rapid shallow breathing index; PFP (cmH_2_O*breaths/min) = pressure frequency product (respiratory frequency x level of pressure support); renal dysfunction= creatinine level≥2mg/dL at weaning; MIP= maximal inspiratory pressure; MIP percentage = percentage of measured MIP in relation to predicted MIP according to age and sex; BNP=B-type natriuretic peptide; Renal ICU= Intensive Care Unit.

**Table 2.** PiCCO hemodynamics according to spontaneous breathing trial outcome before (Pre-T) and following (End-T) the trial.

		**Weaning success (N=14)**	**Weaning failure (N=11)**	**P-value**
MAP, mmHg	Pre-T	86.5 (2.6)	95.2 (2.6)	0.02
	End-T	91.1 (3.2)	100.4 (6.6)	NS
HR, beats/min	Pre-T	92.1 (4.8)	82.82 (5.2)	NS
	End-T	95.9 (4.4)	94.0 (57.6)*	NS
RPP, beats_*_min^−1^_*_mmHg	Pre-T	7974 (502)	7985 (603)	NS
	End-T	8786 (550)	9648 (1072)	NS
ScvO_2_, %	Pre-T	76.6 (1.7)	74.5 (3)	NS
	End-T	79 (1.5)	72.6 (2.5)	0.03
O_2_ER, %	Pre-T	22.8 (1.6)	24.4 (2.9)	NS
	End-T	19.3 (1.5)*	24.3 (2.2)	NS
CVP, mmHg	Pre-T	5.43 (1)	11.27 (1.9)	0.01
	End-T	4.86 (0.9)	10.82 (1.6)	0.01
EVLW, mL	Pre-T	430 (20.4)	603 (56)	0.01
	End-T	440 (30)	566.4 (43)	0.01
ELWI, mL/kg	Pre-T	6.79 (0.7)	9 (0.7)	0.04
	End-T	6.21 (0.6)	8.54 (0.7)	0.01
CI, L/min/m^2^	Pre-T	3.82 (0.3)	3.63 (0.3)	NS
	End-T	4.22 (0.3)*	4.25 (0.4)*	NS
SVI, mL/m^2^	Pre-T	45.3 (3.4)	43.9 (4.8)	NS
	End-T	48.1 (3.3)	47 (4.3)	NS
GEDI, mL/m^2^	Pre-T	659 (19)	846 (75)	0.013
	End-T	691 (31)	861 (80)	0.04
PBV, mL	Pre-T	349.3(12.4)	452 (82.6)	NS
	End-T	367.7(15.9)	410 (45.6)	NS
SVRI, cm.d.sec^−5^m^2^	Pre-T	1833 (108)	2122 (199)	NS
	End-T	1755 (101)	1924 (222)	NS
dPmx, mmHg/min	Pre-T	1357 (118)	1070 (130)	NS
	End-T	1266 (154)	1146 (113)	NS
PVP	Pre-T	1.28 (0.1)	1.65 (0.2)	0.028
	End-T	1.22 (0.1)	1.5 (0.2)	NS
CFI, L/min	Pre-T	5.79 (0.4)	4.58 (0.6)	NS
	End-T	6.16 (0.4)	5.25 (0.6)	NS
GEF, %	Pre-T	52.2 (3.8)*	42.36 (4)*	NS
	End-T	26.7 (0.9)*	23.94 (3)*	NS

Data represent mean±SE otherwise is indicated. P-values represents significant differences (P≤0.05) between weaning success and weaning failure subgroups.

* Significant differences (P≤0.05) between Pre-T and End-T values within subgroups of weaning outcome.

MAP= mean arterial pressure; Pre-T=pre-trial; End-T= end-trial; HR= heart rate; ScvO_2_= caval oxygen saturation; O_2_ER=oxygen extraction ratio; CVP= central venous pressure; EVLW= extravascular lung water; ELWI= extra-vascular lung water, indexed to predicted body weight; CI= cardiac index; SV= stroke volume index; SVI= stroke volume, indexed to body surface area; GEDV= global end-diastolic volume; GEDI= global end-diastolic volume, indexed to body surface area; PBV= pulmonary blood volume; SVRI= systemic vascular resistance index; dPmx= maximum pressure increase in the aorta (ΔPmax/Δt); PVP= pulmonary vascular permeability; PVPI= pulmonary vascular permeability, indexed to body surface area; SVV= stroke volume variation; CFI= cardiac function index; GEF= global ejection fraction RPP= Rate-pressure product.

**Table 3.** Echocardiographic parameters in T-piece weaning trial in respect to T-trial and weaning outcomes.

		**SBT-outcome**			**Weaning Outcome**		
		**Success, n=20**	**Failure, n=5**	**P value**	**Success, n=14**	**Failure, n=11**	**P value**
**Right Ventricular Echocardiography**							
RVFAC, %	Pre-T	44.2 (2.1)	33.32 (16)	-	46.26 (2.2)	37.32(6.5)	-
RV/LV EDA	Pre-T	0.58 (0.04)	0.38 (0.03)	0.02	0.54 (0.04)	0.54(0.06)	-
RV Sm, cm/sec	Pre-T	16.61 (0.8)	13.25 (0.9)	-	17.08 (0.96)	14.9(0.9)	-
	End-T	16.39 (0.9)	14.25 (0.7)	-	16.93 (1.24)	14.9(0.7)	-
RV Em, cm/sec	Pre-T	13.49(1.33)	13.38 (2)	-	14.09 (1.58)	12.3 (1.2)	-
	End-T	12.33(0.93)	12.37 (0.8)	-	13.39 (1.05)	12.3(1.2)	-
RV Am, cm/sec	Pre-T	17.37 (1.5)	18.97 (2.3)	-	16.8 (1.49)	11 (1)	-
	End-T	18.27 (1.1)	22.3 (2.43)	-	18.56 (1.23)	19.1 (1.7)	-
RV IVA, m/sec^2^	Pre-T	5.22 (0.59)	2.97 (0.71)	-	5.76 (0.66)	3.7 (0.5)	0.021
	End-T	4.44 (0.65)	3.21 (0.12)	-	4.93 (0.83)	2.9 (0.15)	0.05
RV Tei index	Pre-T	0.66 (0.08)	0.65 (0.15)	-	0.67 (0.1)	0.66(0.1)	-
	End-T	0.79 (1.13)	0.6 (0.05)	-	0.83 (0.18)	0.64(0.1)	-

**Left Ventricular Echocardiography**							
LVEF, %	Pre-T	62.63 (2.1)	58.22 (8.1)	-	63.14 (2.9)	60 (3.7)	-
LV mass, g/m^2^(Penn)	Pre-T	270 (27)	268 (58)	-	267.9 (39.5)		-
IVSWT, mm	Pre-T	10.8 (0.7)	9.4 (0.6)	-	11 (1)	10 (0.4)	-
LVPWT, mm	Pre-T	9.9 (0.5)	8.7 (0.3)	-	10 (0.7)	9.5 (0.3)	-
LVIDd, mm	Pre-T	45.5 (1)	49.1 (5)	-	44.6 (1.4)	48.2 (1.9)	-
LVIDs, cm	Pre-T	30 (0.7)	34.3 (5.3)		28.7 (0.6)	33.5 (0.2)	0.024
E, cm/sec	Pre-T	72.28 (4.9)	83.3 (8.6)	-	67.18 (3.14)	83.8 (8.3)	0.05
	End-T	80.71(5.7) *	95.1 (8.9 *	-	76.14(3) *	93.1(10) *	-
A, cm/sec	Pre-T	76.98 (4.8)	88.7 (7.4)		70.31 (4.36)	90.2 (6.2)	0.014
	End-T	81.63 (5.2)	97.6 (7.8)	-	78.51 (4.76)	93.6 (7.3)	-
DTE, msec	Pre-T	212.4(14.4)	169.5 (27)	-	198.6 (11.7)	211.6(22)	-
	End-T	190.9 (12)	154.8 (24)	-	179.82 (11)	183(19.5)	-
E/A	Pre-T	0.96 (0.05)	0.97 (0.13)	-	0.96 (0.05)	0.95(0.1)	-
	End-T	1 (0.05)	1.02 (0.17)	-	0.99 (0.04)	1(0.1)	-
LV Sm, cm/sec	Pre-T	11.71 (0.6)	10.27 (1.6)	-	11.42 (0.8)	10.3(0.8)	-
	End-T	11.94 (0.8)	11.95 (1.4)	-	12.98 (1)	10.7(0.8)	-
LV Em, cm/sec	Pre-T	10.07 (0.7)	9.16 (0.9)	-	10.05 (0.6)	9.5 (1)	-
	End-T	11.62(0.9) *	11.1 (1.4) *	-	12.1 (1) *	11 (1.3) *	-
LV Am, cm/sec	Pre-T	9.7 (0.4)	12 (0.9)	0.003	10.08 (0.43)	10.6(0.8)	-
	End-T	11.1 (0.8)	13.24 (1.4)	-	11.22 (1)	11.9 (1)	-
E/Em	Pre-T	7.87 (0.9)	9.56 (1.5)		6.87 (0.35)	9.8 (1.6)	0.05
	End-T	7.85 (0.1)	9.08 (1.32)	-	6.73 (0.6)	9.72 (1.7)	-
LV IVA, m/sec^2^	Pre-T	3.58 (0.39)	2.69 (0.37)	-	3.8 (0.47)	2.6 (0.2)	0.046
	End-T	3.37 (0.4)	2.67 (0.47)	-	3.8 (0.47)	2.5 (0.36)	0.05
LV Tei index	Pre-T	0.64 (0.06)	0.8 (0.15)	-	0.62 (0.09)	0.72(0.1)	-
	End-T	0.71 (0.11)	0.75 (0.2)	-	0.73 (0.17)	0.7 (0.1)	-

Data representmean±SE otherwise is indicated.

* Spontaneous Breatning Trial-induced significant changes within subgroups, examined by paired t-test analysis.

RV=right ventricular; RVFAC= right ventricular fractional area change; LV= left ventricular; EDA= end-diastolic area; RV Sm, Em, Am = Tissue Doppler Imaging (TDI)-derived peak systolic, early and late diastolic velocity, respectively (measured at the lateral border of tricuspid annulus); Pre-T=pre-trial; End-T= end-trial; IVA= isovolumic acceleration velocity; LVEF= Left ventricular ejection fraction; IVSWT= inter-ventricular septum wall thickness; LVPWT= left ventricular posterior wall thickness; LVIDd= left ventricular internal diameter at end-diastole; LVIDs=left ventricular internal diameter at end-systole; E and A= pulsed wave Doppler early and late transmitral left ventricular filling wave velocity, respectively; DTE= E–wave deceleration time; LV Sm, Em, Am = Tissue Doppler Imaging (TDI)-derived peak systolic, early and late diastolic velocity, respectively (measured at the lateral border of mitral annulus).

**Table 4.** Univariate and independent determinants of extravascular lung water index (pre spontaneous breathing trial values)

	**Univariate linear regression analysis**			**Multivariate linear regression analysis**		
	**r**	**R2**	**P value**	**b**	**B (95%CI)**	**P value**
GEF	−0.481	0.231	0.03	−0.013	−0.003 (−0.024 – 0.019)	NS
LV Sm	−0.487	0.237	0.01	0.004	0.004 (−0.131 – 0.139)	NS
LV E/Em	0.404	0.163	0.04	0.203	0.133 (0.063 – 0.202)	0.01
CVP	0.431	0.185	0.03	−0.041	−0.018 (−0.066 – 0.031)	NS

ELWI= extravascular lung water index; r=Pearson’s correlation coefficient; R2= coefficient of determination; B, b= unstandardized and standardized beta coefficients, respectively; CEF=global ejection fraction; LV=left ventricular; Sm, Em= peak systolic and early diastolic tissue-Doppler myocardial velocities at the lateral border of the mitral annulus, respectively; E=E-wave mitral inflow pulsed-wave Doppler velocity; CVP=central venous pressure

### Early outcome (T-trial failure)

Five out of our 25 patients (20%) failed SBT. SBT success was significantly associated with lower Pre-T EVLW (ml) [(478(28) vs 646(115), P=0.04], PBV (ml) values [355(21) vs 578(182), P=0.04].

**Fig. 2.**
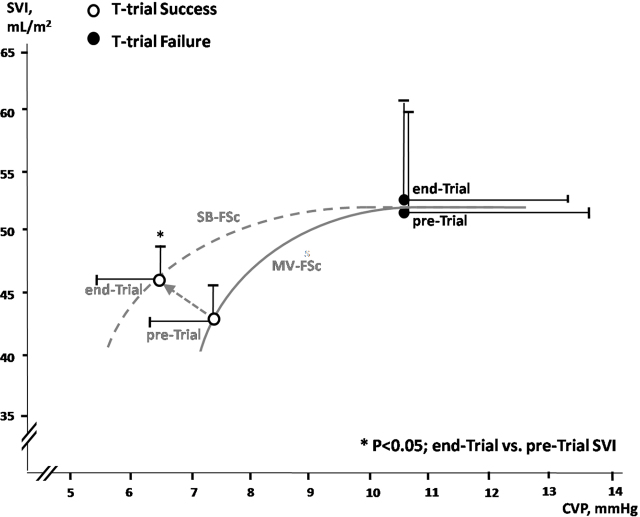
Stroke Volume Index (SVI) against central venous pressure (CVP) plot and Frank Starling curve representations in participants during spontaneous breathing and mechanical ventilation. Open circles represent successful weaning, closed circles represent weaning failure; SB-FSc=Frank-Starling’s curve at spontaneous breathing; MV-FSc= Frank-Starling’s curve during mechanical ventilation.

The change (%Δ) in variables during SBT [(End-T) – (Pre-T)/(Pre-T)] between SBT success and failure patients was significantly different in: cardiac index [10.0(2.4) vs 30.1(5.8), P=0.001], rate-pressure product [9.4(4) vs 45.8(26) P<0.019), oxygen extraction ratio [−16.7(5.3) vs 62.6(12.6), P<0.001], ScvO_2_ [5.33(2.1) vs −14.8(2.1), P<0.001) and systemic vascular resistance [−2.2(2.6) vs −21.7(7.5), P<0.02]. Moreover, SBT failure patients had higher end-trial CVP (cmH_2_0) (Figure 2) and GEDI (mL/m^2^) [715(32) vs 967(144), (P=0.013)] values compared to patients with SBT success.

## Discussion

The major findings of the present study were that weaning outcome of critical patients undergoing SBT was significantly associated a) with ELWI, GEDI and b) impaired diastolic function, as indicated by pre-T E/Em; moreover, c) a remarkable proportion of patients (56%) presented ELWI≥7mL/kg when fulfilling predetermined criteria for weaning. These findings suggest that latent cardiac dysfunction may play a role in weaning and it is likely to discriminate patients not ready-to-wean, still highly-dependent on the ventilator’s external labor.

In the present study, weaning outcome was associated with pre-T-piece trial ELWI values; ELWI is considered a surrogate of pulmonary oedema. Notably, more than half of our patients presented while on assisted ventilation, before the initiation of T-piece trial, ELWI values (≥7mL/kg); these values are considered compatible with increased pulmonary capillary permeability and oedema [12]. Other conventional markers of evaluation of fluid-balance [8,10,13], did not show significant association with weaning outcome. Tables 2.

Interestingly, in this study we found that while patients had increased ELWI values had no clinical signs of fluid overload while on positive airway pressures or at the beginning of T-trial. We believe that positive airway pressures masked edematous lung conditions in those patients. Transition to spontaneous breathing during T-trial and negative intrathoracic pressures revealed gradually this condition of pulmonary oedema and thus, it induced hypoxemia and weaning failure; this might be possibly mediated by transposing interstitial edema towards lung periphery as it has been previously reported [14]. It is well known that positive pressure ventilation may exert a “pleiotropic” beneficial cardiorespiratory impact [15,16] that can result in EVLW redistribution from the subpleural towards the (less crucial for gas-exchange) peribronchial lung areas, improving oxygenation [14]. In clinical practice when such a condition is revealed during weaning, clinicians usually restart positive ventilation and potentially modify their strategy on patient’s fluid balance (indeed that was the main therapeutic option in our patients).

A future study that could assess local redistribution of lung fluid by real time imaging, during the transition to spontaneous breathing, may provide more insight in this issue. Nevertheless, our study suggests that, negative pleural pressure associated pulmonary edema may represent a common, probably underestimated, cause of difficult weaning.

In our study, despite the association between ELWI and weaning outcome, no relationship was found between weaning outcome and ELWI changes (Table 2). There were no significant EVLW changes during the trial, despite the withdrawal from mechanical ventilation and the transition to spontaneous breathing. Moreover, there was no echocardiographic evidence of ensuing cardiac dysfunction in terms of left ventricle ejection function but impaired diastolic function prior to SBT, as indicated by E/Em values and CVP, GEDI. On this basis “weaning-induced” cardiogenic pulmonary edema, defined as acute fluid extravasation within lungs secondarily to weaning-induced cardiac dysfunction [5,17] is not likely to constitute a crucial pathogenic mechanism of weaning failure in our patients.

We noted that patients who failed weaning (or the SBT) had significantly higher pre-trial EVLW values whereas they presented an indication towards EVLW decline during the trial; interestingly, the higher the pre-trial EVLW values, the greater its decline during the trial (r=−0.413, P=0.04). We speculate that ELWI decline during SBT might not represent a paradox. It may represent an “inward” trans-vascular fluid shift ^2^ constituting a physiologic response of redistribution of fluid in the lungs [18,19].. In patients who failed weaning this fluid swift might be not adequate considering the final outcome. We assume that this mechanism could be associated with the intense sympathetic stimulation which is triggered when spontaneous unassisted breathing can’t be tolerated [3,20,21]. We certainly acknowledge that catecholamine levels were not assessed in our study. However, patients who presented SBT failure presented signs compatible with sympathetic over-discharge (greater cardiac index increases, apparently due to overwhelming increases in heart rate). We therefore hypothesize that these hyperdynamic circulatory features, probably mediated by adrenergic over-stimulation, were compensatory responses [17].

In this study, patients had no known pre-existing heart disease. However, weaning outcome was associated with impaired cardiac contractility and diastolic dysfunction whereas patients with SBT failures manifested “high-output heart failure”-like features presenting upslope in cardiac output and oxygen consumption, significant decline in caval-oxygen saturation and also in systemic vascular resistances. Latent diastolic dysfunction may have predisposed to weaning failure; this is in line with our previous study [10]. Diastolic cardiac dysfunction and impaired capillar pulmonary permeability/pulmonary edema may be causally interrelated [3]. Both entities may represent epiphenomena of underlying inflammation and ongoing critical illness [22]. It cannot be assured whether echocardiographic evidence of cardiac dysfunction reflects inflammatory cardiomyopathy or not [23].

There are certain points that have to be accounted when interpreting the results of this study. We aimed to assess by echocardiography the general ICU population who undergo weaning and in this respect, patients with preexisting disorders that could decrease the diagnostic accuracy of echocardiographic assessment were excluded. Similarly, we excluded patients with preexisting cause of neuromuscular diaphragmatic weakness (i.e. myasthenia, muscular dystrophies, spinal cord injury, multiple sclerosis, amyotrophic lateral sclerosis, poliomyelitis, Guillain-Barre syndrome, diaphragmatic of phrenic nerve injury from tumor, trauma, following viral infection or radiation therapy). However patients with previous pulmonary disorders or ARDS were not excluded. Hence, our results may not apply to the general ICU population.

Moreover, the size of our population is small to draw definitive conclusions. We certainly acknowledge that the present was a physiologic study aiming to explore pathophysiologic mechanisms and a large clinical study would be necessary to provide solid answers in this context. However, despite the relatively small population of this study, one might argue that based on GEDI pre-SBT values (the relationship between GEDI values and weaning outcome was one of the main findings in the study) which were significantly different between patients with weaning success and failure [659(19) mL/m^2^ vs 846(75) mL/m^2^, p=0.01 respectively], a post hoc analysis would give a power of 100% (with a statistical alpha value of 0.05).

Another point to be considered is the EVLW cutoff value for pulmonary oedema. Previous studies reported “normal” values for EVLW between 5–7 ml/kg while considerably higher values (as high as 30 ml/kg) have been reported during severe pulmonary edema [7,12,16,24]. In this respect we thought that any value above 7ml/Kg might reflect pulmonary oedema and that value was considered more appropriate as a cutoff in this study. We certainly agree that a higher value such as 10ml/Kg might correspond better to clinically detectable pulmonary oedema.

Moreover, it should be pointed out that ELWI measurement by thermodilution has been used to evaluate pulmonary oedema [12] both in mechanically ventilated [6,25,26,27] and spontaneous breathing patients [4]. One other point for consideration is the use of thermodilution during spontaneous breathing Under such conditions, both acute changes in CO or in V/Q (following mechanical ventilation withdrawal) [28,29,30] may be also have an impact in the accuracy of measurements. We regret we have not included serial measurements or/and a larger population and in our study in order to address these questions in pre-specified groups (i.e. patients with acute CO changes or not, patients with large V/Q disorders or not). This could be the scope of a future study.

## Conclusion

Our findings suggest that increased ELW in non-cardiac critical care patients who fulfill criteria for withdrawal from positive pressure ventilation, are common and may be associated with adverse weaning outcome. In this respect all factors associated with pulmonary oedema (i.e. ELW) should be adequately controlled at spontaneous weaning trials. SBT failure may indicate early respiratory exhaustion due to inadequate cardiac reserve to compensate for the edema-induced increased respiratory workload.
